# An Overview of the Treatment Options Used for the Management of COVID-19 in Pakistan: Retrospective Observational Study

**DOI:** 10.2196/28594

**Published:** 2021-05-27

**Authors:** Hashaam Akhtar, Samar Akhtar, Fazal-Ul Rahman, Maham Afridi, Sundas Khalid, Sabahat Ali, Nasim Akhtar, Yousef S Khader, Hamaad Ahmad, Muhammad Mujeeb Khan

**Affiliations:** 1 Yusra Institute of Pharmaceutical Sciences Yusra Medical and Dental College Islamabad Pakistan; 2 Department of Medicine Benazir Bhutto Hospital Rawalpindi Pakistan; 3 Department of Biotechnology Quaid-i-Azam University Islamabad Pakistan; 4 School of Chemical and Materials Engineering National University of Science and Technology Islamabad Pakistan; 5 Department of Gynecology and Obstetrics Pakistan Air Force Hospital Islamabad Pakistan; 6 Department of Infectious Diseases Pakistan Institute of Medical Sciences Islamabad Pakistan; 7 Medical Education and Biostatistics Department of Community Medicine, Public Health and Family Medicine, Faculty of Medicine Jordan University of Science and Technology Irbid Jordan; 8 Department of Infectious Diseases Rawalpindi Medical University Rawalpindi Pakistan

**Keywords:** COVID-19, antibiotics, Pakistan, multidrug resistant infections, antibiotic resistance, first wave

## Abstract

**Background:**

Since the first reports of COVID-19 infection, the foremost requirement has been to identify a treatment regimen that not only fights the causative agent but also controls the associated complications of the infection. Due to the time-consuming process of drug discovery, physicians have used readily available drugs and therapies for treatment of infections to minimize the death toll.

**Objective:**

The aim of this study is to provide a snapshot analysis of the major drugs used in a cohort of 1562 Pakistani patients during the period from May to July 2020, when the first wave of COVID-19 peaked in Pakistan.

**Methods:**

A retrospective observational study was performed to provide an overview of the major drugs used in a cohort of 1562 patients with COVID-19 admitted to the four major tertiary-care hospitals in the Rawalpindi-Islamabad region of Pakistan during the peak of the first wave of COVID-19 in the country (May-July 2020).

**Results:**

Antibiotics were the most common choice out of all the therapies employed, and they were used as first line of treatment for COVID-19. Azithromycin was the most prescribed drug for treatment. No monthly trend was observed in the choice of antibiotics, and these drugs appeared to be a random but favored choice throughout the months of the study. It was also noted that even antibiotics used for multidrug resistant infections were prescribed irrespective of the severity or progression of the infection. The results of the analysis are alarming, as this approach may lead to antibiotic resistance and complications in immunocompromised patients with COVID-19. A total of 1562 patients (1064 male, 68.1%, and 498 female, 31.9%) with a mean age of 47.35 years (SD 17.03) were included in the study. The highest frequency of patient hospitalizations occurred in June (846/1562, 54.2%).

**Conclusions:**

Guidelines for a targeted treatment regime are needed to control related complications and to limit the misuse of antibiotics in the management of COVID-19.

## Introduction

In December 2019, a viral outbreak of pneumonia was reported in a city in China in December 2019 [1]; this outbreak would have a substantial impact worldwide. Shortly after the first reports of the disease, it rapidly spread globally and was declared a pandemic by the World Health Organization in 2020 [2]. The official name for this pneumonia-like disease is COVID-19, and the virus that causes it is called SARS-COV-2 [3]. The pathological conditions of COVID-19 infection were classified into five categories, from asymptomatic to critical, according to clinical manifestations [4]. It has already been reported that approximately one-fifth of the hospitalized patients with COVID-19 are admitted to intensive care units due to difficulty in breathing or acute hypoxemic respiratory failure [5-8].

More than a year has passed since the initial outbreak, and SARS-CoV-2 continues to spread globally, crippling the economy, damaging health, and causing mortality each day; the current count has reached a staggering 112.65 million confirmed infections and 2.49 million deaths [9,10]. With the rapid spread of COVID-19 across the world, prompt diagnostic tools, readily available repurposable drugs, and effective containment measures to control SARS-CoV-2 infection are of paramount importance. The pandemic has also exposed inadequate research and health infrastructures globally, especially in countries such as Pakistan, where basic health care necessities were scarce when the infection reached its peak in June 2020 [11,12].

The second wave of COVID-19 infections being reported by various countries, including Pakistan, is proving to be even more challenging to address because of the severity of COVID-19–related complications, which vary with gender and age [13-15], underlying diseases and disorders, and even delay in hospital admissions [16,17]. Human behavior is also a major factor causing the resurge in infections [18]. The relationship between adherence to precautions and cases of COVID-19 is clear: in areas where fewer people wear masks and more people gather indoors to eat, drink, celebrate, socialize, and observe religious practices, even if only with family, cases are on the rise [19-21].

With overburdened health care systems and an increasing number of infections among medical staff, the ultimate way to overcome this pandemic remains the discovery of an effective vaccine. Although pharmaceutical companies worldwide have introduced several vaccines to date [22,23], effectively vaccinating a sufficiently large number of people all over the world is a lengthy process [24,25]. Moreover, with reports of new strains from different regions, the effectiveness of some of these vaccines against multiple mutant strains shows mixed results [26]. As a result, repurposing existing drugs to target SARS-CoV-2 and treat COVID-19–associated symptoms still appears to be a logical scientific approach at the moment to contain this pandemic. Identifying and appropriating an effective combination of drugs from the available repertoire is a challenge in itself. The hit-and-trial method is dangerous but inevitable in the current situation. Small-scale studies were performed in which a few drugs were reported to be effective; however, these drugs were later proved to result in no significant difference in clinical outcomes [27-29].

Currently, as observed in various reports globally as well as in Pakistan, supportive treatment, mechanical ventilation, and extracorporeal membrane oxygenation remain the primary treatment choices for medical practitioners. Therapeutic options that are being considered and used include antiviral, antiparasitic, and anti-inflammatory medications; interferon therapy; convalescent plasma therapy; hyperimmunoglobulin; oligonucleotide-based therapies; and, rarely, RNA interference and mesenchymal stem cell therapy [30-32].

In this study, we explored the use of antibiotic and antiviral drugs for the treatment of patients admitted during the peak of the first wave of COVID-19 in Pakistan (May-July 2020). Directions for treatment of the disease during the first wave phase were not very clear, and many necessities, including drugs, were out of stock in local markets due to lockdowns, high demand, limited stocks, and closure of borders.

## Methods

### Study Design

Clinical data from 1812 confirmed patients with COVID-19 admitted to four major tertiary care hospitals in Pakistan, that is, Pakistan Air Force Hospital, Islamabad, Pakistan Institute of Medical Sciences Hospital, Islamabad, Holy Family Hospital, Rawalpindi, and Benazir Bhutto Shaheed Hospital, Rawalpindi, were retrospectively collected during the period from February to August 2020.

### Patient Selection, Timeline, and Data Collection

Confirmed COVID-19 cases were defined as patients with a positive polymerase chain reaction test for COVID-19 from nasal and oropharyngeal swab samples taken at the time of admission to the hospital. Patients with incomplete data were excluded. Descriptive data of 1562 patients admitted during the months of May to July 2020 were abstracted and analyzed accordingly. The data included information about the patients’ age, gender, dates of admission and discharge (or death), medical history, presenting signs and symptoms, initial categorization of COVID-19 (mild, moderate, severe, and critical), and types of therapeutic agents (including but not limited to use of antibiotics, antimalarials, antivirals, antiparasitics, anticoagulants, and corticosteroids) used for treatment and management of COVID-19 during their hospital stay.

The statistical analysis was conducted using SPSS, version 24 (IBM Corporation) and Stata 16.1 (StataCorp LLC). Categorical variables were described using frequencies and percentages [33,34]. Chi-square tests and Fisher exact tests were used to compare percentages wherever appropriate. This retrospective cohort study was approved by the ethics review board of Rawalpindi Medical University. Data were collected with approval of the National Institute of Health (NIH), Pakistan.

## Results

A total of 1562 patients were included in the study; 1064 (68.1%) were male and 498 (31.9%) were female, with a mean age of 47.35 years (SD 17.03). The basic demographic characteristics of the hospitalized patients with COVID-19 and their distribution across the hospitals are shown in Table 1. The frequencies of admission during the months of May, June, and July 2020 were 37.9% (592/1562), 54.2% (846/1562), and 7.9% (124/1562), respectively.

**Table 1:** Baseline characteristics of the study participants (N=1562).

Characteristic			Value
Age (years)**,** mean (SD)			47.35 (17.03)
**Gender**			
	Male	1064 (68.1)	
	Female	498 (31.9)	
**Admission frequency of patients across hospitals, n (%)**			
	Benazir Bhutto Shaheed Hospital, Rawalpindi	813 (52.0)	
	Holy Family Hospital, Rawalpindi	470 (30.1)	
	Pakistan Institute of Medical Sciences Hospital, Islamabad	135 (8.3)	
	Pakistan Air Force Hospital, Islamabad	144 (9.2)	
**Admission frequency of patients across the period of the study, n (%)**			
	May 2020	592 (37.9)	
	June 2020	846 (54.2)	
	July 2020	124 (7.9)	

**Figure 1:**
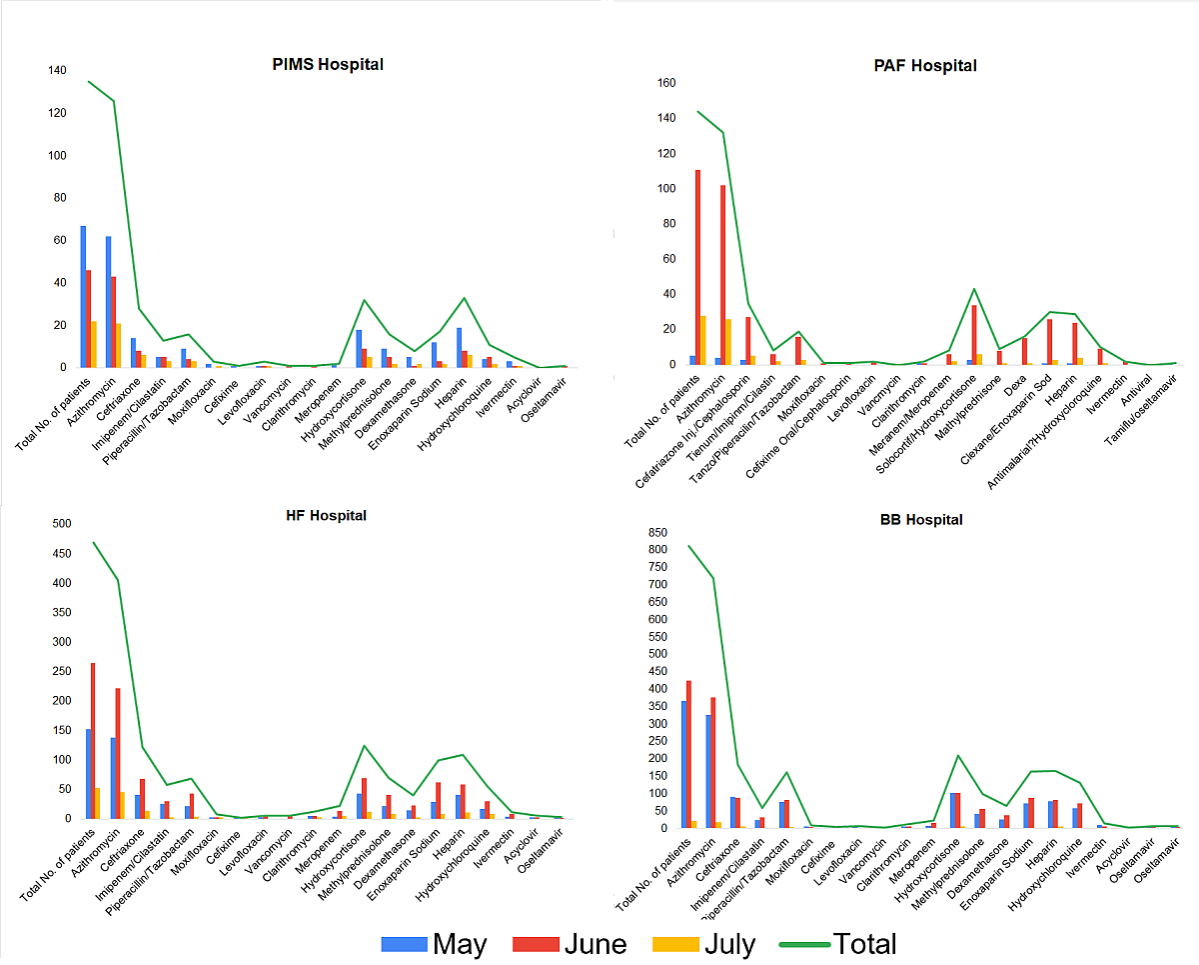
Hospital-wise relative distribution of different drugs administered to patients with COVID-19 during the first wave of the pandemic in Pakistan. BB: Benazir Bhutto; HF: Holy Family; PAF: Pakistan Air Force; PIMS: Pakistan Institute of Medical Sciences.

**Figure 2:**
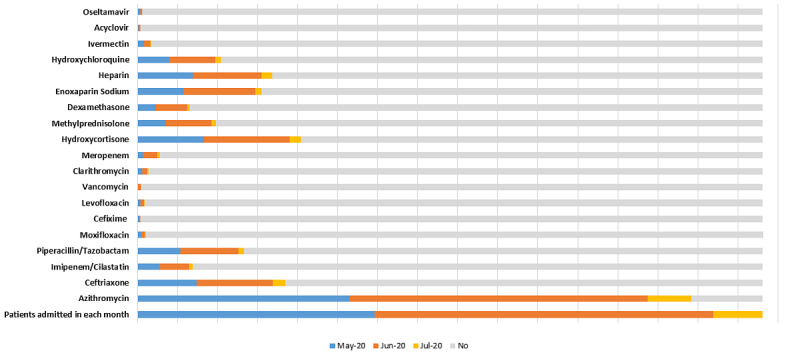
Relative distributions of different drugs administered to patients with COVID-19 in each month during the first wave of the pandemic (May-July 2020) in Pakistan.

Of the 19 drugs reportedly used for treatment of COVID-19 and management of COVID-19–related symptoms, the most frequently used antibiotic was azithromycin (1384/1562, 88.6%), followed by ceftriaxone (369/1562, 23.6%). Anticoagulants such as heparin (337/1562, 21.6%) and enoxaparin sodium (310/1562, 19.8%) and steroids such as hydrocortisone (409/1562, 25.7%) were also among the 5 most frequently used drugs. The relative distribution of administered drugs across hospitals during the first wave of COVID-19 is given in Figure 1. The load of patients at each hospital was different; however, the trends of the regimens used were similar. The peak of the first wave of COVID-19 in Rawalpindi-Islamabad was observed during June 2020. The trends of drug use during the first wave of COVID-19 in Pakistan are shown in Figure 2 and Table 2.

Although the load over hospital varied during the 3 months, the choices of drugs used to treat COVID-19 remained the same. The frequencies and percentages of the prescription of these drugs are given in Table 3. Different combinations were used, and these combinations included drugs from various categories, such as anticoagulants, corticosteroids, and antibiotics.

**Table 2:** Distributions of different drugs administered to patients with COVID-19 (N=1562) in each month during the first wave of the pandemic (May-July 2020) in Pakistan.

Drug	Prescriptions, n (%)			
	Patients admitted in May 2020 (n=592)	Patients admitted in June 2020 (n=846)	Patients admitted in July 2020 (n=124)	Patients not prescribed^a^
Azithromycin	531 (89.7)	743 (87.8)	110 (88.7)	178 (11.4)
Ceftriaxone	148 (25)	191 (22.6)	30 (24.2)	1193 (76.4)
Imipenem/cilastatin	55 (9.3)	74 (8.7)	10 (8.1)	1423 (91.1)
Piperacillin/tazobactam	107 (18.1)	145 (17.1)	14 (11.3)	1296 (83)
Moxifloxacin	11 (1.9)	7 (0.8)	3 (2.4)	1542 (98.7)
Cefixime	4 (0.7)	3 (0.4)	1 (0.8)	1554 (99.5)
Levofloxacin	8 (1.4)	9 (1.1)	1 (0.8)	1544 (98.8)
Vancomycin	1 (0.2)	8 (0.9)	0 (0)	1553 (99.4)
Clarithromycin	12 (2.0)	13 (1.5)	3 (2.4)	1534 (98.2)
Meropenem	14 (2.3)	35 (4.1)	7 (5.6)	1506 (96.4)
Hydrocortisone	166 (28)	214 (25.3)	29 (23.4)	1153 (73.8)
Methylprednisolone	72 (12.2)	113 (13.4)	12 (9.7)	1365 (87.4)
Dexamethasone	46 (7.8)	78 (9.2)	6 (4.8)	1432 (91.7)
Enoxaparin sodium	115 (19.4)	180 (21.3)	15 (12.1)	1252 (80.2)
Heparin	138 (23.3)	172 (20.3)	27 (21.8)	1225 (78.4)
Hydroxychloroquine	79 (13.3)	116 (13.7)	13 (10.5)	1354 (86.7)
Ivermectin	17 (2.9)	16 (1.9)	1 (0.8)	1528 (97.8)
Acyclovir	3 (0.5)	5 (0.6)	0 (0)	1554 (99.5)
Oseltamivir	7 (1.2)	5 (0.6)	0 (0)	1550 (99.2)

^a^Percentages calculated based on the total number of patients across 3 months (N=1562).

**Table 3:** Drugs used during the first wave of COVID-19 to treat patients (N=1562) in all four hospitals under study.

Drug		Value, n (%)
**Antibiotics**		
	Azithromycin	1384 (88.6)
	Ceftriaxone	369 (23.6)
	Cefixime	8 (0.51)
	Meropenem	56 (3.6)
	Imipenem/cilastatin	139 (8.9)
	Piperacillin/tazobactam	266 (17.0)
	Vancomycin	9 (0.6)
	Clarithromycin	28 (1.8)
	Moxifloxacin	21 (1.3)
	Levofloxacin	18 (1.1)
**Corticosteroids**		
	Hydrocortisone	409 (26.2)
	Methylprednisolone	197 (12.6)
	Dexamethasone	130 (8.3)
**Anticoagulants**		
	Enoxaparin sodium	310 (19.8)
	Heparin	337 (21.6)
**Antimalarial**		
	Hydroxychloroquine	208 (13.3)
**Antiparasitic**		
	Ivermectin	34 (2.2)
**Antiviral**		
	Acyclovir	8 (0.5)
	Oseltamivir	12 (0.8)

## Discussion

### Principal Findings

Our results show that the highest proportion of admissions occurred in the month of June (846/1562, 54.2%), just after Eid-ul-Fitr (the Muslim festival, which was held on May 23 and 24 in 2020). It is worth noting that this was the time when the first wave of COVID-19 infections was at its peak in Pakistan; however, the trend of the treatment regimen remained the same during the period of the first wave [35-37]. However, after this period, a dramatic decrease in infections was observed due to effective precautions and regulations imposed by the government, including “smart lockdown” in potential hotspots, implementation of standard operating procedures, and closure of academic buildings [38].

Our study reports the use of up to 10 antibiotics of different classes. The effectiveness of the use of antibiotics for the treatment of COVID-19 is debatable, and evidence of their direct inhibitory effect on viral replication or pathogenesis remains to be proved. These antibiotics are generally used to treat upper respiratory tract infections, pneumonia, and other infections caused by opportunistic bacteria due to low immunity during viral infection. Azithromycin was widely used because it is a broad-spectrum antibiotic and can treat chest infections, including pneumonia, which is also a manifestation of COVID-19 infection; infections of the nose and throat, such as sinus infections (sinusitis); skin infections; Lyme disease; and some sexually transmitted infections [39].

In addition to antibiotics, some other frequently used drugs were anticoagulants (heparin and enoxaparin sodium) and corticosteroids (hydrocortisone and methylprednisolone). Use of anticoagulants and steroids is indicated for reduction of the inflammatory effects of SARS-CoV-2, which helps control disease progression to a limited extent; however, no succinct combination was observed in terms of treating COVID-19 [40].

### Challenges and Shortcomings on the Therapeutic Front

Despite the fact that the antibiotics supported the combined therapies used against COVID-19, there is still no evidence that supports the use of these antibiotics to treat viral infection by health care professionals in Pakistan. Other drugs, such as anticoagulants and steroids, were also used as supportive therapy; however, there is a need to establish standard guidelines to treat patients with COVID-19–related complications. The experimental hit-and-trial approach of various combinations of drugs and the alarming frequency with which antibiotics were used will eventually lead to antibiotic resistance in the human population. One of the major challenges faced by health care professionals globally was the reliability on available therapies against the newly introduced virus. Mutations in RNA viruses are more frequent compared to those in DNA species; COVID-19, being an RNA virus, is a great threat to humanity. Modern research is necessary to study mutable infectious agents to develop multifaceted therapies to target the pathways of infection.

### Conclusion

This study highlights the trend of drugs used to treat COVID-19 infections in early the months of the pandemic across Pakistan. The use of antibiotics by health care professionals to treat COVID-19 is questionable. It signifies the lack of specific guidelines that must be followed by all hospitals in terms of treatment regimens, and organizations such as the NIH and the Centers for Disease Control and Prevention must not only provide guidelines to address the pandemic but also ensure that those guidelines are strictly being followed throughout the country. This study reveals the weaknesses in the health care infrastructure and the inadequacy of hospitals and staff in Pakistan. With the second wave emerging in various countries and a mutant strain of SARS-CoV-2 causing infections, it is important to ensure that the standard operating procedures are being strictly followed, a proper treatment guideline is provided, and drugs used for symptomatic treatment are monitored to avoid antibiotic resistance in the future.

### Limitations of the Study

The study was limited to the Rawalpindi and Islamabad regions of Pakistan, which are large cities with relatively good health care facilities and checks and balances on health care practitioners. Data from other cities, especially small towns and rural regions, can be helpful in analyzing the misuse of medicines prescribed for treatment of COVID-19–related symptoms and complications. The study was performed on patients admitted in mid-2020 during the peak of the first wave, and a better analysis could be performed if data were taken from the months of the second wave as well. Collecting data during the first wave was difficult due to limited access to COVID-19 wards and a shortage of personal protection equipment. The number of subjects included in the study was under 2000; comprehensive information could be gathered from a large population size.

## Abbreviations

NIH: National Institute of Health
